# The Response of Smokers to Health Warnings on Packs in the United Kingdom and Norway Following the Introduction of Standardized Packaging

**DOI:** 10.1093/ntr/ntab027

**Published:** 2021-02-18

**Authors:** Crawford Moodie, Catherine Best, Ingeborg Lund, Janne Scheffels, Nathan Critchlow, Martine Stead, Ann McNeill, Sara Hitchman, Anne Marie Mackintosh

**Affiliations:** 1 Institute for Social Marketing and Health, Faculty of Health Sciences and Sport, University of Stirling, Stirling, UK; 2 Department of Alcohol, Tobacco and Drugs, Norwegian Institute for Public Health, Oslo, Norway; 3 National Addiction Centre, Institute of Psychiatry, Psychology and Neuroscience, King’s College London, London, UK

## Abstract

**Introduction:**

Standardized packaging was phased in between May 2016 and May 2017 in the United Kingdom and July 2017 and July 2018 in Norway. In both countries, the health warnings on packs prior to standardized packaging being implemented were from the former Tobacco Products Directive library of warnings (text warnings covering 43% of the pack front and pictorial warnings covering 53% of the pack reverse). The warnings on packs, postimplementation, were from the current Tobacco Products Directive library of warnings (novel pictorial warnings covering 65% of the pack front and reverse) for the United Kingdom but unchanged in Norway.

**Aims and Methods:**

Longitudinal online surveys were conducted prior to standardized packaging (United Kingdom: April–May 2016; Norway: May–June 2017) and postimplementation (United Kingdom: September–November 2017 and May–July 2019; Norway: August–September 2018). We explored smokers’ response to the on-pack warnings (salience, cognitive reactions, and behavioral reactions).

**Results:**

In the United Kingdom, noticing warnings on packs, reading or looking closely at them, thinking about them, thinking about the health risks, avoidant behaviors, forgoing cigarettes, and being more likely to quit due to the warnings significantly increased from waves 1 to 2, and then decreased from waves 2 to 3, but remained higher than at wave 1. In Norway, noticing warnings, reading or looking closely at them, thinking about them, thinking about the health risks, and being more likely to quit due to the warnings significantly decreased from waves 1 to 2; avoidant behaviors and forgoing cigarettes remained unchanged.

**Conclusions:**

The inclusion of large novel pictorial warnings on standardized packs increases warning salience and effectiveness.

**Implications:**

Two longitudinal online surveys in the United Kingdom and Norway explored the impact of standardized packaging on warning salience and effectiveness. That warning salience and effectiveness only increased in the UK postimplementation, where standardized packaging was implemented alongside new larger pictorial warnings on the pack front and reverse, and not in Norway, where standardized packaging was introduced but older smaller text warnings (pack front) and pictorial warnings (pack reverse) were retained, highlights the importance of removing full branding and introducing stronger warnings simultaneously.

## Introduction

In 2012, Australia became the first country to legally require tobacco products to be sold in standardized (plain) packaging. Fourteen countries have subsequently fully implemented this policy, including at least one country in all WHO regions except Africa. The core aims of standardized packaging are to reduce the appeal of the packaging and smoking, reduce misperceptions of harm as a consequence of pack design, and increase the salience and effectiveness of the on-pack warnings,^1^ which is the focus of this paper.

Prior to standardized packaging being introduced in the United Kingdom, eye-tracking research^2,3^ and naturalistic studies, where smokers used standardized packs for 1 or 2 weeks,^4,5^ found warnings to be more salient and effective on standardized packs than on fully branded packs. These studies provided important insight into eye-movements toward warnings on standardized packs in laboratory settings, and self-reported response to warnings on standardized packs over a short period of time. However, they were not able to offer any understanding as to whether this response would reflect what happens in countries with standardized packaging or be sustained over time.

In the first five countries to have fully implemented standardized packaging (Australia, France, United Kingdom, New Zealand, and Norway), the sell-through period, ie, the time they had to sell remaining fully branded packs, ranged from 2 to 12 months.^6^ Several studies, all in the United Kingdom, explored smokers’ response to the on-pack warnings during this period. Eye-tracking research with daily or weekly smokers found more than twice the number of fixations to warnings on standardized packs than on fully branded packs.^7^ Poundall et al.^8^ showed university students images of fully branded and standardized packs and found that smokers and nonsmokers were more likely to report noticing the warnings on a standardized pack, and indicate that they would put them off smoking. Both studies were conducted before standardized packs were widely available on the market however. An online survey conducted near the end of the sell-through period, when both fully branded and standardized packs were on sale, found that smokers using standardized packs were more likely than those who had never used standardized packs to have noticed and read or looked closely at the warnings.^9^ While these findings suggest that standardized packaging may improve the impact of warnings, at least during the sell-through period, when they are novel, there is a need for pre–post comparisons and cohort designs to explore the effect of habituation.^10^

Several studies have explored smokers’ response to on-pack warnings since standardized packaging has been implemented. Serial cross-sectional telephone surveys in Australia found a significant increase in the proportion of smokers having strong cognitive and emotional responses to warnings and engaging in avoidant behavior 6 months poststandardized packaging.^11^ Yong et al.^12^ found that a year after the introduction of standardized packaging in Australia there was an increase among smokers in looking at warnings before branding on packs, noticing warnings, cognitive reactions to warnings and avoidant behavior, but not in reading warnings or forgoing cigarettes. Using cross-sectional telephone surveys in Australia, research found that compared with smokers recruited prestandardized packaging those recruited 1-year poststandardized packaging were more likely to report noticing warnings, avoid specific warnings when buying tobacco, cover packs, and attribute much motivation to quit to warnings.^13^ Longitudinal surveys in seven European countries (England, Germany, Greece, Hungary, Poland, Romania, and Spain) found that approximately a year after the inclusion of novel pictorial warnings on packs in all countries, as a result of the Tobacco Products Directive (TPD),^14^ the greatest increase in warning salience was in England, the only country in which standardized packaging had been fully implemented.^15^

A limitation of the aforementioned research is that it was not possible to extricate the role of the novel, larger warnings from the removal of full branding.^9,16,17^ This is because in Australia and the United Kingdom, and many countries with standardized packaging, the removal of full branding and inclusion of larger novel warnings are introduced simultaneously. Norway is a notable exception. As part of the European Free Trade Association, Norway is not part of the EU but submits to EU regulations, such as the TPD, although not necessarily at the same time as EU member countries. When Norway implemented standardized packaging, it did so prior to transposing the current TPD into Norwegian law. The current TPD, which was transposed into UK law, requires packs of cigarettes and rolling tobacco to display one of 14 pictorial warnings on 65% of the pack front and reverse.^14^ There are 14 warning messages (eg, “Smoking causes heart attacks”) and three sets of pictorials for each message, with these sets rotated annually. Norway instead retained the warnings required by the former TPD, which stipulated that packs of cigarettes and rolling tobacco had to display one of two text warnings on 30%–35% of the pack front and one of 14 pictorial warnings on 40%–50% of the pack reverse.^16^ All warnings required a black border, which increased the proportion of the pack surfaces covered.^16^ There were 14 warning messages (eg, “Smoking causes fatal lung cancer”) in the former TPD and three different pictorials for each message, but unlike the current TPD countries were not obliged to rotate the pictorials and instead free to select which one of the three pictorials they wanted to accompany each warning message.

**Figure 1.**
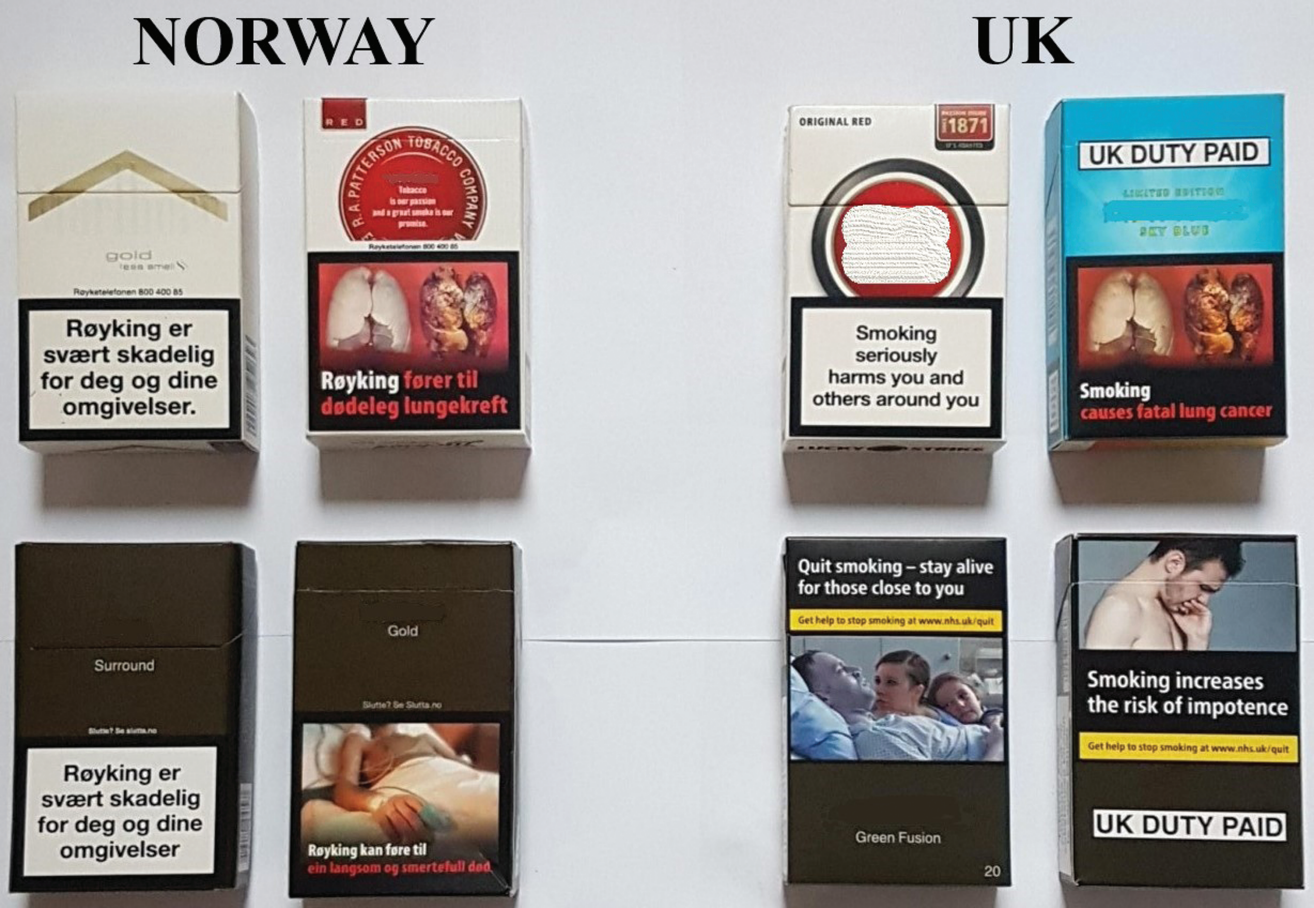
Warnings on packs in Norway and the UK prestandardized packaging (top row) and poststandardized packaging.

In this study, we explored the impact of standardized packaging on warning salience and effectiveness among smokers in the United Kingdom and Norway. In both countries, the warnings prestandardized packaging were the same type (text-only on pack front, pictorial on pack reverse) and size (43% of pack front, 53% of pack reverse), with the same positioning (starting from the bottom of the pack) and very similar content (warning text and images). There was some variation in content, with additional text used for three warnings in the United Kingdom (eg, “You can do it, we can help. Your doctor or pharmacist can help you stop smoking” rather than “Your doctor or pharmacist can help you stop smoking”) and six of the 14 pictorials were different, but otherwise the warnings in both countries were very similar. Poststandardized packaging, the warnings in Norway were unchanged, with the warnings in the United Kingdom taken from the current TPD (novel pictorial warnings starting from the top of the pack and covering 65% of the main display areas), see Figure 1.

## Methods

### Design and Sample

In the United Kingdom, the “Adult Tobacco Policy Survey” is a longitudinal online survey, following a cohort of cigarette smokers recruited prestandardized packaging (April–May 2016) and followed up 4–6 months poststandardized packaging (September–November 2017) and 24–26 months poststandardized packaging (May–July 2019). To be eligible for inclusion at Wave (W) 1, participants had to be 16 or over and report smoking cigarettes (factory-made and/or hand-rolled) in the last 3 months. The sample was recruited from the online panel of YouGov, a market research company. Randomly selected panel members received an e-mail invite to participate and a survey link if they chose to do so. Of the 13 930 invitations sent to panel members whose profiling data suggested they were smokers, 8758 people clicked on the link and 1599 were screened out for not meeting the inclusion criteria. Of the 7159 who started the survey, there were 665 non-completers and YouGov removed 260 participants for data quality issues (eg, straight-lining), leaving 6234 participants. An additional participant was removed by the research team at W2 as their data could not be linked.

Participants at W1 were recontacted at W2 and again at W3, even if they had not participated at W2. Of the 6233 cigarette smokers at W1, 4293 were followed up at W2 (3629 cigarette smokers, 607 ex-cigarette smokers, 36 non-cigarette smokers, 7 cigarette smokers that had not smoked in the past 3 months, 14 missing data on smoking status) and 3175 at W3 (2412 cigarette smokers, 700 ex-cigarette smokers, 44 non-cigarette smokers, 6 cigarette smokers that had not smoked in the past 3 months, 13 missing data on smoking status). Participants received an increased incentive at each wave in an attempt to increase retention: 200 points on their YouGov account (equivalent to £2.00) at W1, 300 points at W2, and 400 points at W3. An information page was provided at the start of the survey, with explicit consent required for participation. The study received ethical approval from the University of Stirling, with the first two waves approved by the Faculty of Health Sciences and Sport Ethics Committee and the third by the General University Ethical Panel.

In Norway, a longitudinal online survey followed a cohort of smokers and snus users prestandardized packaging (May–June 2017) who were followed up 2–3 months poststandardized packaging (August–September 2018). To be eligible for inclusion at W1, participants had to be 16 or over and report smoking cigarettes (factory-made and/or hand-rolled) in the last 3 months. Participants were drawn from the online panel of Kantar TNS. To find eligible participants, Kantar firstly asked individuals about their use of tobacco, with participants then drawn from this pool of tobacco users. We do not have details on the number of participants invited or completion rate. A total of 1665 smokers participated at W1, with 1051 of these responding at W2 (813 smokers, 230 ex-smokers, 4 non-cigarette smokers, 1 had not smoked in the past 3 months, 3 missing). Participants received points on their Kantar account at each wave. The study received ethical approval from the data protection officer at the Norwegian Institute of Public Health.

### Measures

#### Demographics

In the United Kingdom, information was captured on age, gender, household income, and highest educational qualification. Age at W1 was recoded into “16–24,” “25–39,” “40–55,” and “56 and over.” Annual household income was categorized as Low (under £30 000), Medium (£30 000–£44 999), High (£45 000 and over), and Don’t know or prefer not to answer. Highest educational qualification obtained was categorized as Low (High school), Medium (Technical, trade school, A levels, or community college), High (University degree or higher degree), and Don’t know or prefer not to say.

In Norway, information was captured on age, gender, household income, and highest educational qualification. The same age groups were used as for the United Kingdom. Annual household income was categorized as Low (under 600 000 NOK), Medium (600 000–999 999 NOK), High (1 million NOK and over), and Don’t know or prefer not to answer. Education was categorized as Low (High school), Medium (Technical, trade school), High (4 years or more university or college), and Don’t know or prefer not to say.

#### Smoking Status

At W1 participants in the United Kingdom were asked “Which of the following best applies to you? Please note cigarettes refer to those that are factory-made (packet) and also those that are hand-rolled (rolling tobacco). Cigarettes do not include electronic cigarettes or vaping devices.” The response options were “I smoke cigarettes (including hand-rolled) every day,” “I smoke cigarettes (including hand-rolled), but not every day,” “I do not smoke cigarettes at all, but I do smoke tobacco of some kind (e.g. Pipe, cigar or shisha),” “I have stopped smoking completely in the last year,” “I stopped smoking completely more than a year ago,” “I have never been a smoker,” and “Don’t know.” Non-daily cigarette smokers were subsequently asked: “Can we just confirm, how often do you currently smoke cigarettes (either factory-made or hand-rolled)?,” with response options “At least once a week,” “Less than once a week, but at least once a month,” “Less than once a month, but at least once in the last three months,” “I have not smoked cigarettes in the last three months,” and “Don’t know.” Participants were categorized as cigarette smokers if they indicated that they had smoked at least once in the last 3 months. At W2 and W3 the “I have never been a smoker” option was dropped.

In Norway, participants were asked, at both waves, “Which of the following applies best for you?” with response options “I smoke cigarettes or RYO every day,” “I smoke cigarettes or RYO, but not every day,” “I don’t smoke, or I have quit smoking,” and “Don’t know.” Non-daily cigarette smokers were asked “In the last three months, how often have you smoked cigarettes?” with response options “At least once a week,” “Less than once a week, but at least once a month,” “Less than once a month, but at least once in the last three months,” “I have not smoked in the last three months,” and “Don’t know.” Participants were categorized as cigarette smokers if they indicated that they had smoked at least once in the last 3 months.

#### Cigarettes per Day

The number of cigarettes smoked per day was coded as 10 or fewer (coded as 0), 11–20 (coded as 1), 21–30 (coded as 2), and 31 or more (coded as 3). Missing cases were included as a “missing” category.

#### Warning Salience

Participants were asked “In the last 30 days how often, if at all, have you… ‘noticed the warning labels on packs?’ and ‘read or looked closely at the warning labels on packs’ with response options ‘Never’, ‘Rarely’, ‘Sometimes’, ‘Often’, ‘Very often’ and ‘Don’t know’.” Responses were coded as “Often or Very often” versus “Never, Rarely or Sometimes,” with participants responding “Don’t know” excluded from the analysis. For the proportions responding “Don’t know” at each wave, in each country and for each question, see Table 1.

**Table 1.** Numbers and Percentages of Responding “Don’t Know” to Each Measure

	United Kingdom			Norway	
	W1	W2	W3	W1	W2
	*n*	*n*	*n*	*n*	*n*
	%	%	%	%	%
Noticed warnings	74	52	72	32	45
	1.19	0.83	1.16	1.93	4.45
Read warnings	62	37	46	29	27
	0.99	0.59	0.74	1.75	2.67
Thought about warnings	63	56	50	33	24
	1.01	0.90	0.80	1.99	2.37
Warnings risk	65	77	64	44	35
	1.04	1.24	1.03	2.66	3.46
Avoided warnings	87	49	37	41	15
	1.40	1.14	1.53	2.48	1.92
Covered warnings	83	47	31	23	6
	1.33	1.09	1.28	1.39	0.77
Put pack away	86	50	44	28	7
	1.38	1.16	1.82	1.69	0.90
Used a case	81	43	36	21	2
	1.30	1.00	1.49	1.27	0.26
Forgone cigarettes	124	134	122	79	64
	1.99	3.12	3.84	4.78	6.33
Thought about quitting	146	95	74	78	44
	2.34	2.21	3.07	4.72	5.63

#### Cognitive Response to Warnings

Participants were asked “In the last 30 days how often, if at all, did you think about what the warning labels on packs are telling you?” with response options “Never,” “Rarely,” “Sometimes,” “Often,” “Very often,” and “Don’t know.” These were coded as “Often or Very often” versus “Never, Rarely or Sometimes,” with participants responding “Don’t know” excluded from the analysis. In terms of risk perceptions, the wording was slightly different in Norway. In the United Kingdom, participants were asked “To what extent, if at all, have the warning labels on packs made you think about the health risks of smoking?” and in Norway this question was prefixed with “In the last 30 days.” Participants were also asked “To what extent, if at all, do the warning labels on packs make you more likely to quit smoking?” with response options for this and the risk perception question “Not at all,” “A little,” “Somewhat,” “A lot,” and “Don’t know.” Responses were coded as “A lot” versus “Not at all, A little or Somewhat,” with participants responding “Don’t know” excluded from the analysis.

#### Behavioral Reactions to Warnings

To measure avoidant behaviors, participants were asked “In the last 30 days have you done any of the following to avoid looking at the warnings on packs?… ‘Avoided buying packs with particular warnings on them’, ‘Covered the warnings up to avoid looking at them’, ‘Put the pack away to avoid looking at the warning’, and ‘Used a cigarette case or another pack or container to avoid looking at the warnings.’” Response options were “Yes,” “No,” and “Don’t know” for each, coded as “Yes” versus “No,” with participants responding “Don’t know” excluded from the analysis. To assess forgoing cigarettes, they were asked “In the last 30 days how many times, if any, have the warning labels on packs stopped you from having a cigarette when you were about to smoke one?” (Never, Once, A few times, Many times, Don’t know). Responses were coded as “Many times” versus “Never, Once or A few times,” with participants responding “Don’t know” excluded from the analysis.

### Analysis

Data were analyzed using Stata version 15. Categorical outcomes are reported as percentages. Generalized estimating equations were used to examine changes the proportion of smokers who reporting the warning-related outcomes across survey waves. The dependent variables for the generalized estimating equation were binary so were specified as a binomial distribution, with exchangeable correlation structure and robust standard errors. Survey wave was the independent variable and the analyses were adjusted, at each wave, for age group, gender, household income, education, and cigarettes per day. The working correlation structure accounts for correlation among repeated measurements on individuals over time.

## Results

### W1 Sample Characteristics

Almost half the UK (46.4%) and Norway samples (45.0%) were male. In the United Kingdom, 38.4% had at least a University degree, with 31.5% in Norway having 4 or more years of university or college. Most of the UK sample was in the 40–55 age group (32.9%) and most of the Norway sample in the 56 and over age group (45.8%). Further details of the sample characteristics are shown in Tables 2 and 3.

**Table 2.** Characteristics of the Full Sample by Survey Wave and Country

	United Kingdom			Norway	
	Wave			Wave	
	1	2	3	1	2
Education					
Low	2032	1465	1096	720	521
%	32.6	34.1	34.5	43.2	49.6
Medium	1610	991	720	421	142
%	25.8	23.1	22.7	25.3	13.5
High	2396	1693	1270	524	388
%	38.4	39.4	40.0	31.5	36.9
Don’t know or prefer not to say	195	144	89	0	0
%	3.1	3.4	2.8	0	0
Gender					
Male	2889	2006	1519	749	479
%	46.4	46.7	47.8	45.0	45.6
Female	3344	2287	1656	916	572
%	53.7	53.3	52.2	55.0	54.4
Gross household income					
Low	2840	1909	1359	627	172
%	45.6	44.5	42.8	37.7	16.4
Medium	1294	894	664	571	299
%	20.8	20.8	20.9	34.3	28.5
High	969	665	563	243	173
%	15.6	15.5	17.7	14.6	16.5
Don’t know or prefer not to answer	1130	825	589	224	407
%	18.1	19.2	18.6	13.5	38.7
Age group					
16–24	650	181	82	51	15
%	10.4	4.2	2.6	3.1	1.4
25–39	1795	1089	682	299	155
%	28.8	25.4	21.5	18.0	14.8
40–55	2053	1497	1140	553	320
%	32.9	34.9	35.9	33.2	30.5
56 and over	1735	1497	1271	762	561
%	27.8	34.9	40.0	45.8	53.4
Cigarettes per day					
10 or fewer	3391	1850	1217	1008	487
%	54.4	43.1	38.3	60.5	46.3
11–20	2293	1441	947	555	288
%	36.8	33.6	29.8	33.3	27.4
21–30	435	268	208	49	24
%	7.0	6.2	6.6	2.9	2.3
31 or more	114	69	42	12	7
%	1.8	1.6	1.3	0.7	0.7
Missing	0	665	761	41	245
%	0	15.5	24.0	2.5	23.3
Total	6233	4293	3175	1665	1051
%	100	68.9	50.9	100	63.1

**Table 3.** Characteristics of Cigarette Smokers by Survey Wave and Country

	United Kingdom			Norway	
	Wave			Wave	
	1	2	3	1	2
Education					
Low	2032	1271	859	720^a^	414^b^
%	32.6	35.0	35.6	43.2	50.9
Medium	1610	837	535	421^a^	104^b^
%	25.8	23.2	22.2	25.3	12.8
High	2396	1407	948	524^a^	295^b^
%	38.4	38.8	39.3	31.5	36.3
Don’t know or prefer not to say	195	114	70	0^a^	0^b^
%	3.1	3.1	2.9	0	0
Gender					
Male	2889	1687	1153	749	356
%	46.4	46.5	47.8	45.0	43.8
Female	3344	1942	1259	916	457
%	53.7	53.5	52.2	55.0	56.2
Gross household income					
Low	2840	1657	1058	627^c^	131^d^
%	45.6	45.7	43.9	37.7	16.1
Medium	1294	743	506	571^c^	224^d^
%	20.8	20.5	21.0	34.3	27.6
High	969	549	407	243^c^	126^d^
%	15.6	15.1	16.9	14.6	15.5
Don’t know or prefer not to answer	1130	680	441	224^c^	332^d^
%	18.1	18.7	18.3	13.5	40.8
Age group					
16–24	650	132	57	51^e^	4^f^
%	10.4	3.6	2.4	3.1	0.5
25–39	1795	867	486	299^e^	114^f^
%	28.8	23.9	20.2	18.0	14.0
40–55	2053	1287	876	553^e^	257^f^
%	32.9	35.5	36.3	33.2	31.6
56 and over	1735	1343	992	762^e^	438^f^
%	27.8	37.0	41.1	45.8	53.9
Cigarettes per day					
10 or fewer	3391	1850	1215	1008^g^	487^h^
%	54.4	51.0	50.4	60.5	59.9
11–20	2293	1441	947	555^g^	288^h^
%	36.8	39.7	39.3	33.3	35.4
21–30	435	268	208	49^g^	24^h^
%	7.0	7.4	8.6	2.9	3.0
31 or more	114	69	42	12^g^	7^h^
%	1.8	1.9	1.7	0.7	0.9
Missing	0	1	0	41^g^	7^h^
%	0	0.03	0	2.5	0.9
Total	6233	3629	2412	1665	813
%	100	58.2	38.7	100	48.8

^a^W1 Norway vs. W1 United Kingdom, sig different chi square = 577.8921, *p* < .001.

^b^W2 Norway vs. W2 United Kingdom, sig different chi square = 103.9543, *p* < .001.

^c^W1 Norway vs. W1 United Kingdom, sig different chi square = 138.1678, *p* < .001.

^d^W2 Norway vs. W2 United Kingdom, sig different chi square = 301.7732, *p* < .001.

^e^W1 Norway vs. W1 United Kingdom, sig different chi square = 272.1966, *p* < .001.

^f^W2 Norway vs. W2 United Kingdom, sig different chi square = 100.7198, *p* < .001.

^g^W1 Norway vs. W1 United Kingdom, sig different chi square = 211.7629, *p* < .001.

^h^W2 Norway vs. W2 United Kingdom, sig different chi square = 62.8587, *p* < .001.

In the United Kingdom, 68.9% of the W1 sample responded at W2 and 50.9% at W3, and in Norway 63.1% of the W1 sample responded at W2. Young people were more likely to be lost to follow up, with this effect greater in the United Kingdom than in Norway. People with lower levels of education were a higher proportion of the Norway sample at baseline but had a higher probability of being retained in the UK sample.

### Warning Salience

#### How Often Have You Noticed the Warnings?

In the United Kingdom, 25.2% reported noticing the warnings often or very often at W1. This increased to 40.4% at W2 (adjusted odds ratio [AOR] = 2.13; 95% confidence interval [CI] 1.98–2.29) and remained significantly higher at W3 (31.5%) than at W1 (AOR = 1.48; 95% CI 1.36–1.62). There was a significant decrease between W2 and W3 (OR = 0.69; 95% CI 0.63–0.76). In Norway, the proportion noticing the warnings often or very often significantly decreased from W1 (34.7%) to W2 (27.4%) (AOR = 0.67; 95% CI 0.56–0.80).

#### How Often Have You Read or Looked Closely at the Warnings?

In the United Kingdom, 8.6% reported reading or looking closely at warnings often or very often at W1. This increased to 16.5% at W2 (AOR = 2.27; 95% CI 2.03–2.53) and remained significantly higher at W3 (12.5%) than at W1 (AOR = 1.68; 95% CI 1.47–1.92). There was a significant decrease between W2 and W3 (AOR = 0.74; 95% CI 0.65–0.84). In Norway, the proportion reading the warnings often or very often significantly decreased from W1 (12.0%) to W2 (8.0%) (AOR = 0.69; 95% CI 0.52–0.93).

### Warning Cognitions

#### How Often Do You Think About What the Warnings Are Telling You?

In the United Kingdom, 9.0% reported thinking about warnings often or very often at W1. This increased to 14.7% at W2 (AOR = 1.87; 95% CI 1.68–2.08) and remained significantly higher at W3 (12.4%) than at W1 (AOR = 1.56; 95% CI 1.37–1.77). There was a significant decrease between W2 and W3 (AOR = 0.84; 95% CI 0.74–0.95). In Norway, the proportion thinking about the warnings often or very often significantly decreased from W1 (18.7%) to W2 (13.4%) (AOR = 0.69; 95% CI 0.52–0.93).

#### To What Extent Do the Warnings Make You Think About the Risks of Smoking?

In the United Kingdom, 6.7% reported that the warning made them think about the risks of smoking a lot at W1. This increased to 8.9% at W2 (AOR = 1.51; 95% CI 1.33–1.72) and remained significantly higher at W3 (7.4%) than at W1 (AOR = 1.27; 95% CI 1.08–1.48). There was a significant decrease between W2 and W3 (AOR = 0.84; 95% CI 0.72–0.98). In Norway, the extent to which warnings make participants more likely to think about the risks a lot decreased from W1 (12.0%) to W2 (7.6%) (AOR = 0.56; 95% CI 0.42–0.75).

#### To What Extent Do Warnings Make You More Likely to Quit?

In the United Kingdom, 2.3% of smokers said that warnings made them a lot more likely to quit at W1. This significantly increased to 3.5% at W2 (AOR = 1.59; 95% CI 1.27–1.99), but there was no difference at W3 (2.6%) than at W1 (AOR = 1.25; 95% CI 0.95–1.65). There was also no difference between W2 and W3 (AOR = 0.79; 95% CI 0.60–1.04). In Norway, the extent to which warnings made participants more likely to quit a lot significantly decreased from W1 (8.1%) to W2 (5.1%) (OR = 0.64; 95% CI 0.44–0.92).

### Behavioral Reactions to Warnings

#### Avoided Warnings

In the United Kingdom, 2.3% reported avoiding warnings at W1. This increased to 3.4% at W2 (AOR = 1.72; 95% CI 1.38–2.15) and remained significantly higher at W3 (2.7%) than at W1 (AOR = 1.34; 95% CI 1.02–1.77). There was no difference between W2 and W3 (AOR = 0.78; 95% CI 0.59–1.03). In Norway, there was no difference from W1 (2.6%) to W2 (2.5%) (AOR = 0.84; 95% CI 0.48–1.44).

#### Covered Warnings

In the United Kingdom, 8.8% reported covering warnings at W1. This increased to 16.4% at W2 (AOR = 2.19; 95% CI 1.96–2.44) and remained significantly higher at W3 (12.0%) than at W1 (AOR = 1.56; 95% CI 1.36–1.78). There was a significant decrease between W2 and W3 (AOR = 0.71; 95% CI 0.62–0.81). In Norway, there was no difference from W1 (5.2%) to W2 (4.6%) (AOR = 0.88; 95% CI 0.60–1.31).

#### Put Pack Away

In the United Kingdom, 10.2% reported putting the pack away to avoid the warnings at W1. This increased to 19.3% at W2 (AOR = 2.32; 95% CI 2.10–2.56) and remained significantly higher at W3 (14.6%) than at W1 (AOR = 1.71; 95% CI 1.52–1.94). There was a significant decrease between W2 and W3 (AOR = 0.74; 95% CI 0.66–0.83). In Norway, there was no difference from W1 (7.8%) to W2 (5.6%) (AOR = 0.77; 95% CI 0.55–1.09).

#### Used a Case

In the United Kingdom, 4.3% reported using a case at W1. This increased to 12.1% at W2 (AOR = 2.95; 95% CI 2.59–3.36) and remained significantly higher at W3 (9.5%) than at W1 (AOR = 2.24; 95% CI 1.92–2.62). There was a significant decrease between W2 and W3 (AOR = 0.76; 95% CI 0.66–0.87). In Norway, there was no difference from W1 (4.6%) to W2 (4.4%) (AOR = 0.87; 95% CI 0.59–1.30).

#### Forgoing a Cigarette

In the United Kingdom, 9.4% reported forgoing a cigarette at W1. This increased to 11.7% at W2 (AOR = 1.37; 95% CI 1.23–1.53) and remained significantly higher at W3 (10.9%) than at W1 (AOR = 1.32; 95% CI 1.16–1.50). There was no difference between W2 and W3 (AOR = 0.98; 95% CI 0.85–1.10). In Norway, there was no difference from W1 (17.7%) to W2 (13.8%) (AOR = 0.85; 95% CI 0.68–1.05).

## Discussion

In the United Kingdom, there was an increase in warning salience, cognition, and behavioral reactions from W1 (prestandardized packaging) to W2 (shortly after standardized packaging and the new health warnings were fully implemented), and a decrease from W2 to W3 (approximately 2 years poststandardized packaging) but with responses remaining higher than at W1. In Norway, there was a decrease in warning salience and cognition from W1 (prestandardized packaging) to W2 (shortly after standardized packaging was fully implemented).

In markets with standardized packaging, it has not been possible to know whether increased warning salience and effectiveness is a result of the large novel warnings, the removal of full branding, or both.^9,17^ However, recent longitudinal research in multiple European countries has offered valuable insight into how removing full branding alongside the introduction of stronger (larger novel pictorial) warnings, rather than just introducing stronger warnings, helps increase warning salience.^15^ Our findings complement this study by demonstrating the benefits, in terms of improving warning salience and effectiveness, of introducing standardized packaging and stronger warnings simultaneously rather than introducing standardized packaging while retaining weaker (smaller, old text on front and pictorial on reverse) warnings.

That the warnings on standardized packs in the United Kingdom were new, displayed coloured pictorial images on the main display areas (rather than just the pack reverse), had images that were rotated annually, started from the top of the pack (rather than the bottom), and covered a greater proportion of the main display areas (65% front and reverse compared with 43% front and 53% pack reverse), helps explain the findings.^1,18–26^ In Norway, in contrast, the warnings were unchanged, with the two small text warnings on the pack front, the most visible surface, having been on packs for approximately 7 years by W2. That smokers in Norway would be even more desensitized to the warnings at W2 than at W1 may help to explain why salience and cognitive response to the warnings significantly decreased. It may also explain why behavioral reactions (avoidant behavior and forgoing cigarettes) remained unchanged between waves—smokers were engaging less with the warnings and so had less reason to conceal them or smoke less as a result of them.

The findings from the third wave in the United Kingdom, conducted approximately 2 years poststandardized packaging, show that warning salience and effectiveness remained significantly higher than at the first wave (with the exception of warnings making them want to quit a lot, which remained higher but not significantly so), suggesting this response is sustained over time. However, a decline in warning salience and effectiveness was observed during the poststandardized packaging waves, which is consistent with research showing that people get used to the presence or content of warnings.^27–29^ While with adolescent smokers and nonsmokers rather than adult smokers, cross-sectional school surveys in Australia found that compared with baseline (prestandardized), approximately 5 years poststandardized packaging there were no significant differences in attending to, reading, or talking about, warnings, and a decline in thinking about warnings.^30^ Whether there will be a similar trend in Britain (Scotland, Wales, and England) is not clear given that a new set of 13 warnings, taken from warnings used in Australia since 2002, will be required for packs placed on the market after January 2021, as part of the *Tobacco Products and Nicotine Inhaling Products (Amendment etc.) (EU Exit) Regulations*^31^; the current warnings will remain on packs in Northern Ireland as part of the Northern Ireland Protocol.

The findings should be considered in light of a number of limitations. Limitations associated with the sampling design mean that the findings cannot necessarily be generalized to the wider population of smokers. The samples were drawn from online panels. As participants are not necessarily selected onto these panels using probabilistic sampling we cannot be certain that these panels are representative of smokers in each country.^32,33^ Furthermore, online administration means that populations that are more likely to lack Internet access (eg, the elderly, those on the lowest incomes) may be under-represented. The surveys also under-represent younger smokers. In addition, our findings are reliant on self-report. Attrition is also a problem with longitudinal research,^34^ with approximately half (49%) the UK sample lost by W3 and 37% of the Norway sample by W2. In our samples, young people (under 24 years) were more likely to be lost to follow up, particularly in the United Kingdom. This differential attrition is likely to bias the effect in the direction of underestimating the impact of the warnings as younger adults are more likely to have greater warning salience, and/or cognitive and behavioral response to warnings.^35–37^

For countries moving toward standardized packaging our findings suggest that combining this with large novel pictorial warnings is the optimal approach.

## Supplementary Material

A Contributorship Form detailing each author’s specific involvement with this content, as well as any supplementary data, are available online at https://academic.oup.com/ntr.

ntab027_suppl_Supplementary_Taxonomy_Form
