# Corrigendum to: The Response of Smokers to Health Warnings on Packs in the United Kingdom and Norway Following the Introduction of Standardized Packaging

**DOI:** 10.1093/ntr/ntab061

**Published:** 2021-05-03

**Authors:** Crawford Moodie, Catherine Best, Ingeborg Lund, Janne Scheffels, Nathan Critchlow, Martine Stead, Ann McNeill, Sara Hitchman, Anne Marie Mackintosh

*Nicotine & Tobacco Research* (2021). DOI: 10.1093/ntr/ntab027

In the originally published version of this manuscript, there was an error in the Funding section. The Funding paragraph should read: “The first two waves in the UK were funded by Cancer Research UK and the British Heart Foundation (grant no. A18507). The third wave was conducted by the Public Health Policy Research Unit (PH-PRU), commissioned and funded by the National Institute for Health Research Policy Research Programme. The views expressed are those of the authors and not necessarily those of the NHS, the National Institute for Health Research, the Department of Health and Social Care or its arm’s length bodies, and other Government Departments. The study in Norway was funded by the Norwegian Institute of Public Health.” This has been corrected online.

